# Prognostic Significance of Lung Immune Prognostic Index at Diagnosis in Stage III Non-Small Cell Lung Cancer

**DOI:** 10.3390/curroncol33010043

**Published:** 2026-01-13

**Authors:** Tülay Eren, Engin Eren Kavak, İsmail Dilli, Esra Zeynelgil

**Affiliations:** Department of Medical Oncology, Ankara Etlik City Hospital, 06170 Ankara, Türkiye; engineren2000@yahoo.com (E.E.K.); drdilli78@hotmail.com (İ.D.); esra23.05@hotmail.com (E.Z.)

**Keywords:** NSCLC, stage 3, LIPI score, overall survival, prognostic factor

## Abstract

**Objective**: The aim of this study was to assess the association between the Lung Immune Prognostic Index (LIPI) measured at diagnosis and both event-free survival (EFS) and overall survival (OS) in patients with stage III non-small cell lung cancer (NSCLC). **Methods**: This retrospective cohort included patients diagnosed with stage III NSCLC between September 2022 and July 2024, all of whom had a minimum follow-up duration of six months. LIPI was calculated using the derived neutrophil-to-lymphocyte ratio (dNLR) and lactate dehydrogenase levels at diagnosis. Clinical, demographic, and treatment-related data were systematically collected. Survival outcomes were estimated using the Kaplan–Meier approach, while factors associated with prognosis were examined through Cox proportional hazards models. **Results**: The study population consisted of 68 patients, predominantly male (86.8%), with a mean age of 63.4 ± 8.7 years. According to the Lung Immune Prognostic Index classification, 29 patients (42.6%) were categorized as having a good score, 27 (39.7%) as intermediate, and 12 (17.6%) as poor. During a median follow-up of 15.4 months, a total of 40 progressions and 22 deaths occurred. Median EFS was 17.7, 9.4, and 5.8 months for good, intermediate, and poor LIPI groups, respectively (*p* < 0.001). Median OS was 25.7 months in the good LIPI group, was not reached in the intermediate group due to insufficient events, and was 6.7 months in the poor group (*p* < 0.001). In multivariate Cox analysis, poor LIPI was independently associated with inferior survival (EFS: HR = 2.87, 95% CI: 1.85–4.46, *p* < 0.001; OS: HR = 2.59, 95% CI: 1.40–4.78, *p* = 0.002). **Conclusions**: LIPI calculated at diagnosis is an independent prognostic factor for both EFS and OS in stage III NSCLC. Validation in larger, prospective cohorts is warranted to further define its prognostic role in stage III NSCLC.

## Simple Summary

Patients with stage III non-small cell lung cancer receive different combinations of surgery, chemotherapy, and radiotherapy, yet their outcomes vary widely. Identifying simple markers that help predict prognosis remains an important clinical need. The Lung Immune Prognostic Index is based on routine blood tests that reflect inflammation and tumor metabolism. In this study, we evaluated whether this index, calculated at the time of diagnosis, could predict survival outcomes in patients with stage III lung cancer. We found that patients with higher Lung Immune Prognostic Index scores had significantly shorter survival and higher risk of disease progression. These findings suggest that routinely available blood parameters may help identify high-risk patients early in the disease course. Future studies may use this information to improve risk stratification and guide clinical trial design in locally advanced lung cancer.

## 1. Introduction

Lung cancer is the most common cause of cancer-related death globally, with non-small cell lung cancer (NSCLC) representing approximately 85% of all diagnosed cases [1,2,3]. Nearly 25% of patients are diagnosed with stage III lung cancer, which represents a biologically diverse and clinically complex subgroup that continues to experience limited long-term survival despite the use of multimodality treatment strategies [4,5]. Current management strategies—including surgery, chemoradiation, and perioperative systemic therapy—have improved survival but remain insufficient for accurate risk stratification in clinical practice [6,7].

Traditional prognostic factors such as the TNM stage, ECOG performance status, and PD-L1 expression provide valuable information but do not fully capture the complex interactions between tumor burden, systemic inflammation, and host immune response [8,9,10]. Hence, there is an increasing need for simple and reproducible biomarkers that can better reflect the biological heterogeneity of NSCLC and guide therapeutic decision-making.

The Lung Immune Prognostic Index (LIPI) is an inflammation-based composite marker that incorporates serum lactate dehydrogenase (LDH) levels and the derived neutrophil-to-lymphocyte ratio (dNLR) [11]. Elevated LDH reflects tumor hypoxia and high metabolic activity, whereas a high dNLR indicates systemic inflammation and impaired cellular immunity. LIPI classifies patients into good, intermediate, and poor prognostic categories based on these two variables. The LIPI was originally developed to stratify outcomes in patients receiving immune checkpoint inhibitors and has been shown to reflect the interaction between systemic inflammation, tumor metabolism, and antitumor immune response. In the era of immunotherapy, LIPI has therefore gained particular attention as a biomarker associated with treatment response and survival under immune checkpoint blockade. Initially described by Mezquita et al. in advanced NSCLC treated with immune checkpoint inhibitors, the LIPI score has since demonstrated prognostic relevance across several malignancies and treatment settings [12,13].

However, data on the prognostic significance of LIPI specifically at the time of diagnosis in stage III NSCLC remain limited. Understanding its utility in this population is increasingly important in an era where multimodal therapy—including chemoradiation followed by consolidation immunotherapy—is the standard of care. Therefore, this study aimed to evaluate the prognostic impact of baseline LIPI—an index originally developed in the immunotherapy setting—on event-free survival (EFS) and overall survival (OS) in patients with stage III NSCLC.

## 2. Materials and Methods

### 2.1. Study Design and Patient Population

This retrospective cohort analysis comprised patients with histologically confirmed stage III non-small cell lung cancer (NSCLC) who were managed at our center between September 2022 and July 2024. Eligible cases were identified using the institutional oncology registry. Ethical approval for the study was obtained in accordance with institutional regulations and the principles of the Declaration of Helsinki.

### 2.2. Inclusion and Exclusion Criteria

Eligible patients met the following criteria:

(1) Histopathologically confirmed diagnosis of NSCLC;
(2) Clinical stage III disease confirmed by PET-CT imaging according to the 8th edition of the AJCC staging system;
(3) Available baseline laboratory data including lactate dehydrogenase and complete blood counts for calculation of the derived neutrophil-to-lymphocyte ratio (dNLR); and
(4) A minimum follow-up period of 6 months.

Patients with active infection, autoimmune disease, chronic inflammatory conditions, or missing baseline laboratory data were excluded. Patients with missing baseline LDH or CBC data were excluded from LIPI calculation; no imputation was performed.

### 2.3. Calculation of the Lung Immune Prognostic Index (LIPI)

All laboratory measurements were obtained at diagnosis and within 7 days before treatment initiation, and the LIPI score was calculated using LDH and dNLR values recorded at the time of diagnosis. LIPI was calculated using laboratory values obtained at the time of diagnosis in order to capture baseline tumor-related systemic inflammation and metabolic status prior to any treatment-related or supportive-care-related alterations. This approach was chosen to ensure consistency with the original LIPI validation studies and to evaluate the prognostic value of LIPI as a purely baseline biomarker. LDH levels were categorized according to the institutional upper limit of normal (ULN, 220 U/L). The dNLR was calculated as the ratio of neutrophil count to leukocyte count minus the neutrophil count. Based on these parameters, patients were categorized into three prognostic groups:

**Good LIPI**: normal LDH and low dNLR (<3);

**Intermediate LIPI**: one abnormal parameter (either elevated LDH or high dNLR);

**Poor LIPI**: both elevated LDH (≥220 U/L) and high dNLR (≥3).

### 2.4. Data Collection

Demographic data (age, sex, smoking history, comorbidities, ECOG performance status), tumor characteristics (histological subtype, stage, PD-L1 expression, molecular profile), and treatment modalities (surgery, chemotherapy, radiotherapy, immunotherapy) were retrieved from electronic medical records. Radiological assessments were performed using standardized RECIST 1.1 criteria. Comorbidity scores were calculated using the Charlson Comorbidity Index.

### 2.5. Outcome Measures

Overall survival (OS) was designated as the primary outcome and was calculated from the date of diagnosis until death from any cause or the last recorded follow-up. Event-free survival (EFS) was considered the secondary outcome and was measured from the start of treatment to the occurrence of disease progression, recurrence, or death, whichever came first. Patients who remained alive and progression-free were censored at the time of their last follow-up.

### 2.6. Statistical Analysis

Statistical analyses were performed using BlueSky Statistics software (version 10.3.2). The proportional hazards assumption was evaluated by examining Schoenfeld residuals. Continuous variables were summarized using either mean ± standard deviation or median with interquartile range, depending on data distribution, while categorical variables were reported as counts and percentages. Comparisons between groups were conducted using the chi-square test or Fisher’s exact test for categorical variables and the Student’s *t*-test or Mann–Whitney U test for continuous variables, as appropriate.

Time-to-event outcomes were analyzed using the Kaplan–Meier method, and survival distributions were compared with the log-rank test. Cox proportional hazards regression models were applied to determine independent predictors of event-free survival (EFS) and overall survival (OS). Disease stage (IIIA vs. IIIB–C) was incorporated as a covariate in multivariable analyses. Variables demonstrating statistical significance in univariate analyses (*p* < 0.05) were subsequently included in the multivariable models. A two-sided *p*-value of less than 0.05 was considered indicative of statistical significance.

## 3. Results

### 3.1. Patient Characteristics

A total of 68 patients with stage III NSCLC were included in the analysis. The mean age was 63.4 ± 8.7 years, and the majority were male (86.8%) and current or former smokers (92.6%). Adenocarcinoma was present in 33.8% of patients, while 66.2% had squamous cell carcinoma. ECOG performance status was 0–1 in 83.8% of the cohort.

Most patients presented with stage IIIA (45.6%), followed by IIIB (36.8%), and IIIC (17.6%) disease. Lymph node involvement was observed in 91.2% of patients. Only 25% were resectable at diagnosis, and the median SUVmax value was 17.1 (SD 7.4).

The baseline characteristics of the study cohort are summarized in Table 1.

### 3.2. LIPI Distribution and Treatment Patterns

Based on the LIPI classification, 42.6% (*n* = 29) of patients were categorized as good, 39.7% (*n* = 27) as intermediate, and 17.6% (*n* = 12) as poor. The majority of patients (72.1%) received non-surgical treatment. Among 19 surgically treated patients, 63.2% underwent thoracotomy, and 36.8% underwent video-assisted thoracoscopic lobectomy. Negative surgical margins were achieved in 63.2% of resected cases.

The most commonly used systemic chemotherapy regimen was carboplatin–paclitaxel (48.5%), followed by cisplatin–gemcitabine (13.3%). Chemoradiation was performed in 76.6% of patients, most often with weekly carboplatin–paclitaxel. A total of 16 patients (23.4%) did not undergo radiotherapy. Among them, 2 patients declined both chemotherapy and radiotherapy, 2 patients refused radiotherapy while accepting systemic therapy, and 12 patients opted for upfront surgery without neoadjuvant or adjuvant chemoradiation. These choices were primarily patient-driven rather than due to clinical ineligibility. No patient received durvalumab consolidation therapy due to reimbursement limitations and restricted real-world access during the study period, despite its established role following chemoradiation in unresectable stage III NSCLC. Median follow-up was 15.4 months (range 9.4–31.3). During follow-up, recurrence occurred in 58.8% of patients, predominantly with distant metastases (72.5%). This treatment pattern reflects real-world constraints rather than deviation from contemporary guideline-based recommendations.

Treatment details and outcomes are presented in Table 2.

### 3.3. Event-Free and Overall Survival

Across the whole study population, median event-free survival was estimated at 13.5 months (95% CI, 9.9–17.1), whereas median overall survival reached 25.7 months (95% CI, 13.6–37.8).

**Figure 1:**
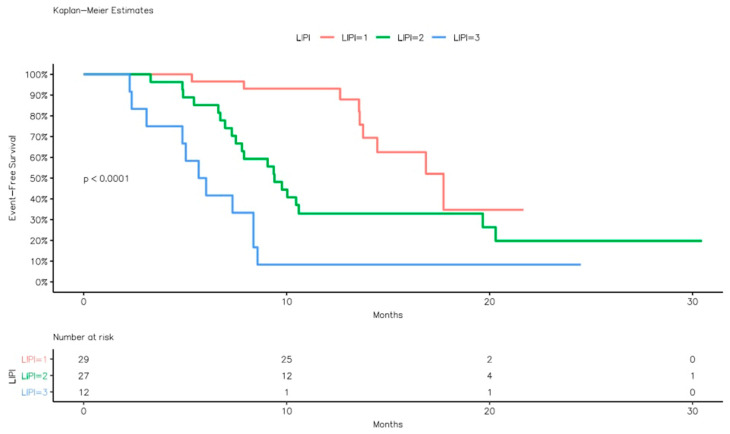
Event-free survival Kaplan–Meier curves according to LIPI groups.

When stratified by LIPI score, patients in the good LIPI group had a median EFS of 17.7 months (95% CI: 14.4–NR) compared with 9.4 months (95% CI: 7.8–20.3) in the intermediate group and 5.8 months (95% CI: 4.8–NR) in the poor group (log-rank *p* < 0.001) (Figure 1).

**Figure 2:**
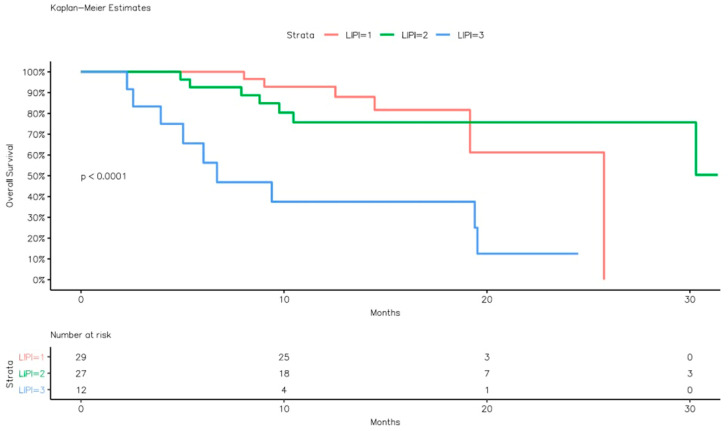
Overall survival Kaplan–Meier curves according to LIPI groups.

Similarly, the median OS was 25.7 months (95% CI: 19.1–NR) for the good LIPI group, not reached for the intermediate group, and 6.7 months (95% CI: 5.0–NR) for the poor LIPI group (log-rank *p* < 0.001) (Figure 2).

### 3.4. Univariate and Multivariate Analysis

In univariate Cox regression analysis, poor LIPI, ECOG ≥ 2, and non-surgical management were significantly associated with worse survival outcomes.

In the multivariate model, LIPI score remained an independent prognostic factor for both EFS and OS after adjusting for ECOG performance status, stage, and surgical treatment.

For EFS, poor LIPI was associated with a 2.41-fold higher risk of progression (HR = 2.41; 95% CI: 1.35–4.28; *p* = 0.003).

For OS, poor LIPI predicted a 2.28-fold higher risk of death (HR = 2.28; 95% CI: 1.21–4.31; *p* = 0.011).

ECOG ≥ 2 also retained prognostic significance for OS (HR = 1.82; 95% CI: 1.09–3.04; *p* = 0.021), whereas stage and surgical status were not independently associated with survival.

The results of univariate and multivariate analyses are summarized in Table 3.

## 4. Discussion

The present study demonstrates that the LIPI, calculated at diagnosis, is a strong and independent prognostic marker for both EFS and OS in patients with stage III NSCLC treated predominantly with definitive chemoradiation. Patients with poor LIPI scores experienced significantly worse survival, and the index remained independently predictive even after adjustment for ECOG status, stage, and treatment modality. The median OS was not reached in the intermediate LIPI group, which is most likely attributable to the limited number of death events and the relatively short follow-up duration rather than a true survival advantage. Therefore, this finding should be interpreted with caution and does not imply superior long-term outcomes compared with the good LIPI group. These findings indicate that baseline systemic inflammation and metabolic stress, captured by LIPI, have a major impact on outcomes in locally advanced disease.

LIPI was originally developed in advanced NSCLC patients receiving immune checkpoint inhibitors by Mezquita et al. [11], and was subsequently validated across different malignancies and treatment settings [12,13]. Our findings are in line with these studies, indicating that LIPI reflects a fundamental biological interplay between tumor metabolism, systemic inflammation, and host immunity, which influences tumor progression regardless of treatment modality or disease stage.

The biological rationale behind LIPI involves two components: LDH, a marker of tumor burden and metabolic activity [13,14], and the derived neutrophil-to-lymphocyte ratio, which reflects systemic inflammation and impaired antitumor immunity [15,16]. Elevated LDH is associated with tumor hypoxia, increased glycolytic metabolism, and aggressive tumor biology, all of which have been linked to resistance to both chemotherapy and radiotherapy [14,15]. Hypoxic tumors are less sensitive to radiation-induced DNA damage and may exhibit reduced chemotherapy efficacy due to altered drug penetration and cellular stress responses.

Similarly, systemic inflammation reflected by a high dNLR has been associated with impaired immune surveillance, reduced lymphocyte-mediated tumor control, and inferior responses to cytotoxic treatments [16,17]. Collectively, these mechanisms provide a biological rationale for the prognostic relevance of LIPI even in patients treated with chemotherapy and radiotherapy, independent of immunotherapy. While these mechanisms have been comprehensively discussed in earlier research, our findings reinforce that LIPI captures a clinically meaningful biological signal even in stage III NSCLC.

The management of unresectable stage III NSCLC has evolved significantly with the introduction of consolidation durvalumab after chemoradiation (the PACIFIC regimen) [18]. Although durvalumab was not used in our cohort, identifying high-risk patients at baseline remains crucial in this modern therapeutic landscape. LIPI may help stratify patients who are less likely to benefit from standard CRT alone and who may require closer surveillance, treatment escalation, or enrollment in clinical trials. Additionally, given its established prognostic performance in immunotherapy-treated metastatic NSCLC [11] and SCLC [19], future studies should evaluate whether LIPI also has predictive value for response to consolidation immunotherapy.

Given the immunotherapy-based origin of the LIPI score, the absence of durvalumab consolidation in our cohort should be considered when interpreting the prognostic implications of LIPI in this setting. Importantly, we do not propose the use of LIPI to guide deviations from the current standard-of-care PACIFIC regimen in routine clinical practice. Rather, LIPI should be viewed as a prognostic and risk stratification tool that may help identify patients at higher risk of poor outcomes who could benefit from closer monitoring or enrollment in clinical trials exploring emerging immunotherapy agents or alternative consolidation strategies beyond durvalumab.

From a clinical perspective, LIPI offers several advantages. It is inexpensive, reproducible, and based on routine laboratory parameters that are universally available. Incorporating LIPI into baseline evaluation could help identify high-risk patients who might benefit from closer monitoring, intensified multimodal treatment, or enrollment in clinical trials exploring novel agents such as consolidation immunotherapy. Furthermore, LIPI may assist in stratifying patients in future clinical trials to minimize prognostic heterogeneity between study arms.

Raphael et al. proposed an expanded “LIPI-based predictive score” in advanced NSCLC, demonstrating that patients with poor LIPI derive limited benefit from immunotherapy alone but may achieve improved outcomes with chemo-immunotherapy combinations [20]. This aligns with our findings that poor-LIPI patients had significantly inferior survival despite multimodal management, suggesting that baseline systemic inflammation and metabolic stress could compromise treatment efficacy regardless of modality. These results collectively emphasize that LIPI is not merely prognostic but may also possess predictive relevance, guiding therapeutic intensity selection in future protocols.

Overall, our study adds novel evidence to a limited set of data examining LIPI specifically in stage III NSCLC. The clear gradient of outcomes across LIPI categories highlights its potential role as a baseline prognostic tool in multidisciplinary decision-making. Prospective multicenter studies, integration with other immune-inflammatory markers, and dynamic assessment during treatment may further enhance its clinical utility. Preliminary findings from this study were previously presented in abstract form as a poster at the European Lung Cancer Congress (ELCC) 2025 [21].

However, this study has several limitations. First, the requirement for a minimum follow-up of six months may have excluded patients with very early mortality and introduced selection bias. The retrospective design and relatively small sample size further limit the statistical power, particularly for subgroup analyses. Treatment regimens were not completely uniform, and 16 patients (23.5%) declined radiotherapy due to personal preference, contributing to real-world heterogeneity that may have influenced survival outcomes. Additionally, key biomarkers such as PD-L1 expression, TMB, and circulating inflammatory markers were not assessed, which may have provided deeper biological insight. Finally, none of the patients received consolidation durvalumab, which is currently considered standard of care after definitive chemoradiation. This represents an important limitation, particularly given that the LIPI score was originally developed and validated in populations treated with immune checkpoint inhibitors. Therefore, our findings should be interpreted in the context of patients treated predominantly with chemoradiation, outside the immunotherapy setting. Despite these limitations, the consistency of our results and their concordance with the existing literature support the robustness of our conclusions.

Overall, our findings support the potential prognostic value of the LIPI score in patients with stage III NSCLC treated with chemotherapy with or without radiotherapy and surgery, outside of the context of immunotherapy.

Future prospective, multicenter studies with larger cohorts are warranted to validate the prognostic role of LIPI specifically in stage III NSCLC. Additionally, exploring dynamic changes in LIPI during treatment may reveal its potential utility as an early marker of treatment response or resistance.

## 5. Conclusions

In conclusion, this study demonstrates that the LIPI is a significant and independent prognostic factor for both event-free and overall survival in patients with stage III NSCLC treated with chemoradiation. These findings highlight the prognostic relevance of baseline systemic inflammation and metabolic status in locally advanced disease, outside the context of immunotherapy. Given its simplicity, accessibility, and strong prognostic value, LIPI may serve as a valuable adjunct to conventional staging systems for risk stratification and treatment planning in locally advanced NSCLC.

## Figures and Tables

**Table 1:** Baseline Characteristics.

Age_Mean ± S.D. Years	63.4 ± 8.7
Sex_*n* (%)	
Female	9 (13.2)
Male	59 (86.8)
Smoking_*n* (%)	
Current or former smoking	63 (92.6)
No smoking history	5 (7.4)
Package/year_median (range)	51.4 (38.3)
Comorbidity score_*n* (%)	
None	24 (35.3)
1	25 (36.8)
2	11 (16.2)
≥3	8 (11.8)
ECOG performance status	
0–1	57 (83.8)
≥2	11 (18.2)
NSCLC histologic type_*n* (%)	
Adenocarcinoma	23 (33.8)
Squamous cell carcinoma	45 (66.2)
Stage_*n* (%)	
IIIA	31 (45.6)
IIIB	25 (36.8)
IIIC	12 (17.6)
Driver Mutation_*n* (%)	
NONE/Not study	63 (92.6)
EGFR	3 (4.4)
Krasg12c	1 (1.5)
Krasg12x	1 (1.5)
PDL-1	
0	47 (69.1)
5≥	9 (13.2)
5–50	9 (13.2)
≥50	3 (4.4)
Lymp Node_*n* (%)	
Positive	62 (91.2)
Negative	6 (8.8)
Resectable_*n* (%)	
Yes	17 (25.0)
No	51 (75.0)
Suvmax_mean ± S.D.	17.1 (7.4)
NLR_mean ± S.D.	2.8 ± 1.4
NLR	
<3	48 (70.6)
≥3	20 (29.4)
LDH(U/L)_mean ± S.D.	223.4 ± 86.7
LDH(U/L)_*n* (%)	
<220	37 (54.4)
≥220	31 (45.6)
LIPI score__*n* (%)	
Good	29 (42.6)
Intermediate	27 (39.7)
Poor	12 (17.6)

Abbreviations: NSCLC, non–small cell lung cancer; ECOG, Eastern Cooperative Oncology Group; LDH, lactate dehydrogenase; LIPI, Lung Immune Prognostic Index; NLR, neutrophil-to-lymphocyte ratio; SUVmax, maximum standardized uptake value; EGFR, epidermal growth factor receptor; KRAS, Kirsten rat sarcoma viral oncogene homolog; PD-L1, programmed death-ligand 1.

**Table 2:** Treatment characteristics and survival outcomes.

Surgical/Nonsurgical approach_*n* (%)	
Surgery only	2 (2.9)
Neoadjuvant Systemic therapy + surgery	2 (2.9)
Surgery + Adjuvant Systemic therapy	12 (17.6)
Surgery+ chemoradiation therapy	3 (4.4)
Definitive chemoradiation therapy (dCRT) only	11 (16.2)
dCRT + Systemic therapy	14 (20.6)
Induction Systemic therapy + dCRT	24 (35.3)
Surgery_*n* (%)	
Thoracoscopic Lobectomy (VATS)	7 (36.8)
Thoracotomy	12 (63.2)
Surgery_*n* (%)	
Lobectomy	13 (68.4)
Pneumonectomy	6 (31.6)
Surgery margin_*n* (%)	
Negative	12 (63.2)
Positive	7 (36.8)
Systemic therapy_*n* (%)	
Carboplatin–Paklitaxel	33 (48.5)
Cisplatin–Gemsitabin	9 (13.3)
Cisplatin–Dosetaxel	2 (2.9)
Cisplatin–Pemetrexet	1 (1.5)
Recurrence_*n* (%)	
Yes	40 (58.8)
No	28 (41.2)
Recurrence_*n* (%)	
Locoregional	11 (27.5)
Distant	29 (72.5)
EFS_median (95% CI) months	13.5 (9.9–17.1)
Treatment after recurrence_*n* (%)	
Yes	28 (70.0)
No (BSC)	12 (30.0)
Immunotherapy after recurrence_*n* (%)	
Yes	20 (72.7)
No	8 (27.3)
Exitus_*n* (%)	
Yes	22 (32.4)
No	46 (67.6)
OS_median (95% CI) months	25.7 (13.6–37.8)
Median Follow-up_months (Range)	15.4 (9.4–31.3)

Abbreviations: CRT, chemoradiation therapy; dCRT, definitive chemoradiation therapy; EFS, event-free survival; OS, overall survival; VATS, video-assisted thoracoscopic surgery.

**Table 3:** Univariate and multivariate Cox regression analysis for EFS and OS.

Variable	Univariate HR (95% CI)	*p*-Value	Multivariate HR (95% CI)	*p*-Value
**EFS**				
LIPI (poor vs. good/intermediate)	2.87 (1.85–4.46)	**<0.001**	2.41 (1.35–4.28)	**0.003**
ECOG (≥2 vs. 0–1)	1.94 (1.16–3.26)	**0.012**	1.58 (0.92–2.71)	0.094
Stage (IIIB–C vs. IIIA)	1.42 (0.86–2.33)	0.172	1.33 (0.79–2.25)	0.275
Surgery (no vs. yes)	1.66 (0.96–2.88)	0.066	1.36 (0.78–2.39)	0.272
**OS**				
LIPI (poor vs. good/intermediate)	2.59 (1.40–4.78)	**0.002**	2.28 (1.21–4.31)	**0.011**
ECOG (≥2 vs. 0–1)	1.97 (1.12–3.47)	**0.018**	1.82 (1.09–3.04)	**0.021**
Stage (IIIB–C vs. IIIA)	1.48 (0.87–2.54)	0.155	1.39 (0.81–2.42)	0.220
Surgery (no vs. yes)	1.57 (0.88–2.79)	0.127	1.36 (0.76–2.45)	0.296

Abbreviations: HR, hazard ratio; CI, confidence interval; EFS, event-free survival; OS, overall survival; ECOG, Eastern Cooperative Oncology Group performance status; LIPI, Lung Immune Prognostic Index. Cox regression analyses were performed for variables with *p* < 0.05 in univariate analysis.

## Data Availability

The data presented in this study are available from the corresponding author upon reasonable request.
